# Warfare: Rallying Around the Environmental Flag

**DOI:** 10.1289/ehp.115-a128

**Published:** 2007-03

**Authors:** John Manuel

Social scientists have long studied competition for natural resources as a source of conflict around the world, but they have paid little attention to the environment in post-conflict societies. Must the environment invariably suffer in the wake of conflict? Can former combatants rally around the environment to help sustain peace? These were the types of questions asked at a November 2006 workshop convened by the Nicholas Institute for Environmental Policy Solutions at Duke University in conjunction with the Environmental Change and Security Program of the Woodrow Wilson International Center for Scholars, the Harrison Program on the Future Global Agenda at the University of Maryland, and the Center for Unconventional Security Affairs at the University of California, Irvine.

Noting that half of all peace agreements collapse within five years, Erika Weinthal, an associate professor of environmental policy at the Nicholas School, asked whether the environment is being addressed in these agreements and whether it can be used to help people think and act “beyond borders.” Ken Conca, director of the Harrison Program on the Future Global Agenda, commented that the UN is involved in 30 peace-keeping missions around the world. One common element of the conflicts he has analyzed is a high environmental toll. “The environmental dimension of peace is ignored at great peril, especially in poor countries, in rural areas, and among disenfranchised people,” Conca said. “Do not look at the environment as a secondary issue to be dealt with later.”

Richard Matthew, director of the Center for Unconventional Security Affairs, cautioned that many factors rule against addressing environmental issues in the immediate wake of conflict. He said that governing bodies charged with protecting the environment typically do not function well or face large budget cuts, while NGOs find it difficult to operate. Infrastructure is often damaged, and criminal activity and profiteering (such as illegal logging) proliferate. Large numbers of refugees and/or internally displaced persons tax natural resources, and traumatized citizenry lack trust in the motives of outside organizations.

As a consultant to the European Commission on peace settlements in Eastern Europe, Alexander Carius of Adelphi Research agreed with Matthew’s summation. “It’s difficult to push transboundary environmental programs when the institutions don’t exist to carry them out,” he said.

At the same time, people in postconflict societies are desperate for water, fuel, shelter, and food. Matthew said every effort should be made to meet these needs in ways that are sustainable from the outset in order to avoid long-term problems that might undermine reconstruction efforts. One example of this is a program sponsored by the Belgian government that supplies gas cookstoves in Rwanda to limit illicit woodcutting.

Liz McBride, director of the Post-Conflict Development Initiative of the International Rescue Committee, said her organization traditionally provided direct relief for victims of war, but eventually recognized the need to be more involved in contributing to durable solutions from the start. “We were good at saving lives, but we needed to strengthen institutions in a way that would bring people back together,” she said. “For us, that now involves building governance systems, which is a movement beyond traditional humanitarian action.” Several speakers acknowledged that in postconflict societies, environmental problems will not be solved unless and until basic governance systems are in place.

Judy Oglethorpe, director of community conservation at the World Wildlife Fund, said her organization recognizes the importance of maintaining a presence throughout a conflict, especially during periods of political transition. “Great windows of opportunity open up when a new government comes in,” Oglethorpe said. “If you are there, you can have an influence on new policies.” She cited as two examples Nepal, where conservation measures have been incorporated into a new draft constitution, and Mozambique, where demobilized soldiers were employed as park guards following that country’s peace agreement in 1994.

Weinthal says the event cosponsors are now trying to come up with a research design to address a range of questions that arose in the wake of the panel and workshop. “We need to come up with recommendations for research that are both theoretical with respect to the environment and have direct policy relevance,” she said.

## Figures and Tables

**Figure:**
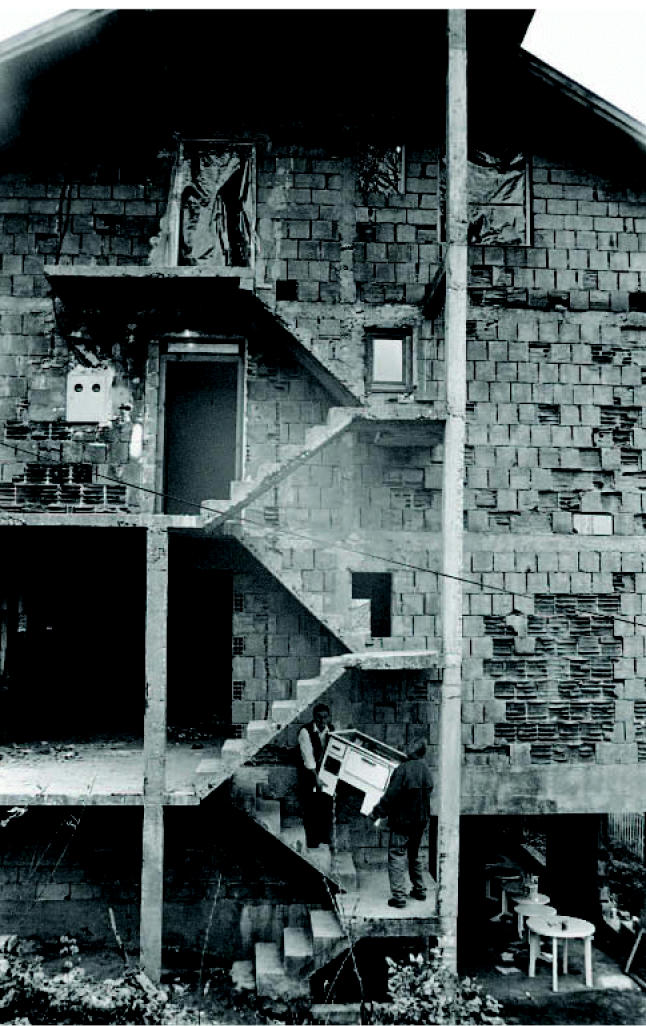
Recovering worlds torn apart Bosnian Muslims return to their homes in Foca, the first to return to the village eight years after an ethnic cleansing campaign by Serbian forces in 1992.

